# [^18^F]FDG PET/CT in the Evaluation of Melanoma Patients Treated with Immunotherapy

**DOI:** 10.3390/diagnostics13050978

**Published:** 2023-03-04

**Authors:** María Mangas Losada, Leonardo Romero Robles, Alejandro Mendoza Melero, Irene García Megías, Amós Villanueva Torres, Puy Garrastachu Zumarán, Xavier Boulvard Chollet, Egesta Lopci, Rafael Ramírez Lasanta, Roberto C. Delgado Bolton

**Affiliations:** 1 Department of Diagnostic Imaging (Radiology) and Nuclear Medicine, University Hospital San Pedro and Centre for Biomedical Research of La Rioja (CIBIR), 26006 Logroño, Spain; 2 Nuclear Medicine, IRCCS Humanitas Research Hospital, 20089 Rozzano, Italy

**Keywords:** FDG PET/CT, immunotherapy, melanoma, response evaluation, adverse events

## Abstract

Immunotherapy is based on manipulation of the immune system in order to act against tumour cells, with growing evidence especially in melanoma patients. The challenges faced by this new therapeutic tool are (i) finding valid evaluation criteria for response assessment; (ii) knowing and distinguishing between “atypical” response patterns; (iii) using PET biomarkers as predictive and response evaluation parameters and (iv) diagnosis and management of immunorelated adverse effects. This review is focused on melanoma patients analysing (a) the role of [^18^F]FDG PET/CT in the mentioned challenges; (b) the evidence of its efficacy. For this purpose, we performed a review of the literature, including original and review articles. In summary, although there are no clearly established or globally accepted criteria, modified response criteria are potentially appropriate for evaluation of immunotherapy benefit. In this context, [^18^F]FDG PET/CT biomarkers appear to be promising parameters in prediction and assessment of response to immunotherapy. Moreover, immunorelated adverse effects are recognized as predictors of early response to immunotherapy and may be associated with better prognosis and clinical benefit.

## 1. Introduction

Melanoma is one of the most aggressive tumours, presenting the highest global growth rate worldwide. The incidence of skin melanoma has increased consistently in fair-skinned people over the past 40 years. It is a challenging tumour that requires a multidisciplinary approach, in which nuclear medicine has a relevant role, including sentinel node biopsy and [^18^F]FDG PET/CT as part of the standard of care [1,2]. Regarding [^18^F]FDG PET/CT, there is extensive evidence showing its efficacy in staging melanoma patients with advanced disease [3]. [^18^F]FDG PET/CT has very high efficacy for detecting distant metastases, but it has limitations when evaluating the presence of microscopic disease in lymph nodes. Therefore, in the initial stages, [^18^F]FDG PET/CT is not useful for lymph node staging, but, in advanced disease with increased tumour burden, [^18^F]FDG PET/CT does detect lymphatic spread [2,3].

On the other hand, immunotherapy, which is based on regulation of the immune system, has been a great advancement in recent decades in the field of oncological diseases. Given the immunogenic nature of melanoma, it is one of the tumours in which immunotherapy is proving more useful. Due to the relatively recent discovery of immunotherapy, there are still challenges that must be clarified, such as (a) finding valid evaluation criteria for response assessment; (b) knowing and distinguishing between “atypical” response patterns; (c) using PET biomarkers as predictive and response evaluation parameters as well as diagnosis and management of immunorelated adverse effects [4]. For all, [^18^F]FDG PET/CT seems to be a useful tool given its ability to study metabolism of lesions and provide information that would not be obtained if based exclusively on morphological changes. However, these processes present as metabolic patterns not evidenced with conventional treatments. High-quality evidence is required to validate the role of [^18^F]FDG PET/CT in this setting and, for this, harmonization of the procedure is needed in order to make results comparable between centres and in different time points [5]. The recently published guidelines on recommended use of [^18^F]FDG PET/CT in solid tumours undergoing immunotherapy [6] have, therefore, become a valuable tool for adequate integration and reporting of this imaging modality in melanoma patients [7,8].

The aim of this review is to analyse the role of [^18^F]FDG PET/CT in the evaluation of melanoma patients treated with immunotherapy, focusing on the main challenges, such as response assessment interpretation criteria, differentiating between “atypical” response patterns and the role of PET biomarkers in this setting.

## 2. Materials and Methods

This is a non-systematic review of articles focusing on the utility of [^18^F]FDG PET/CT in melanoma patients treated with immunotherapy. The inclusion criteria were (a) original and review articles on [^18^F]FDG PET/CT in melanoma patients treated with immunotherapy; (b) published in scientific journals written in English and (c) including at least 20 melanoma patients. Exclusion criteria were (a) full article not available in English and (b) case reports and conference abstracts were not included.

**Figure 1:**
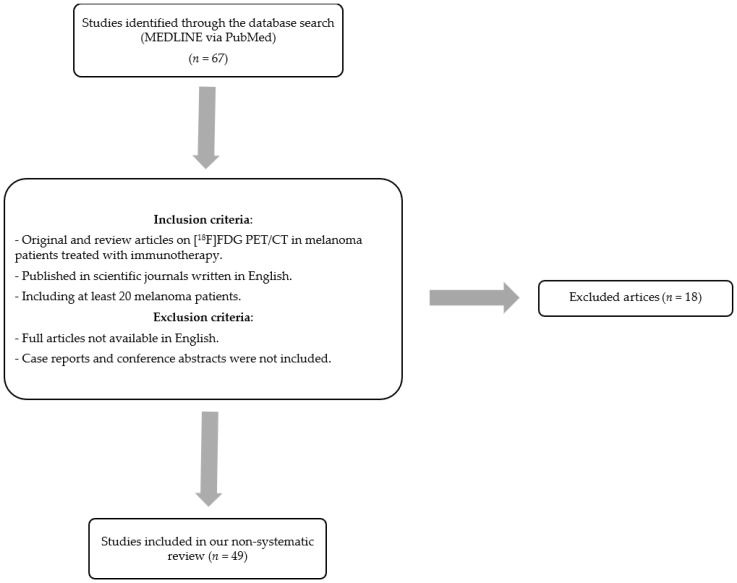
Flow chart of study selection.

One of the selected articles is a recent meta-analysis on this topic, published in 2020 [4]. The articles included in this meta-analysis were included in our systematic review, updating the literature search until the end of 2022. A flow chart of study selection is presented in Figure 1.

## 3. Evaluation Criteria for Response Assessment

Traditionally, since [^18^F]FDG PET/CT for assessment of response in solid tumours has been implemented, multiple clearly defined parameters have been created with good clinical correlation and in terms of overall survival. The need to standardize the evaluation criteria in order to be able to apply them in clinical practice has fuelled in the last 20 years the development of a harmonized and reproducible approach to response evaluation, with the proposal of several new criteria for evaluation and interpretation issues, such as EORTC (European Organization for Research and Treatment of Cancer) [9] and PERCIST (Positron Emission tomography Response Criteria in Solid Tumors) [10], which are commented on in more detail below. Regarding evaluation of response to immunotherapy, it presents different imaging characteristics compared to conventional chemotherapy, and, therefore, with its implementation in clinical practice, there was a need to standardize the evaluation criteria in order to be able to apply them in clinical practice. The most relevant criteria are summarized below.

In 1999, EORTC established four criteria to report the observed results for evaluation of metabolic response that served as the basis for subsequent evaluations after initiation of treatment with a good clinical correlation. These criteria were (a) progressive metabolic disease (PMD) is classified as an increase in [^18^F]FDG tumour SUV greater than 25% within the defined tumour region in the initial scan, visible increase in the extent of [^18^F]FDG tumour uptake (20% in longest dimension) or new [^18^F]FDG uptake in metastatic lesions. (b) Stable metabolic disease (SMD) is classified as an increase in tumour [^18^F]FDG SUV of less than 25% or a decrease of less than 15% and no visible increase in [^18^F]FDG uptake tumour extent (20% in longest dimension). (c) Partial metabolic response (PMR) is classified as a reduction of a minimum of 15 ± 25% in tumour [^18^F]FDG SUV after one cycle of chemotherapy and greater than 25% after more than one treatment cycle. (d) Complete metabolic response (CMR) is classified as complete resolution of [^18^F]FDG uptake within the tumour volume so that it was indistinguishable from surrounding normal tissue [9].

In 2009, PERCIST criteria published by Wahl et al., also included four metabolic categories. EORTC and PERCIST showed high agreement in different types of cancers despite the different approaches of each one. One of the main differences with the EORTC criteria is that PERCIST recommends using SUL instead of SUV, considering lean body mass instead of weight for the calculation. PERCIST criteria establish four categories: (a) CMR defined as disappearance of all metabolically active lesions; (b) PMR is considered for SULpeak reduction ≥30% in the hottest target lesions; (c) SMD is applied when it is neither PMD nor PMR/CMR and (d) PMD is applied when SULpeak increases ≥30% in the hottest target lesion and apparition of new lesions [10].

These two evaluation criteria guidelines, EORTC and PERCIST, serve as the basis upon which the following evaluation criteria have been developed. In 2017, the PECRIT criteria (PET/CT Criteria for early prediction of Response to Immune checkpoint inhibitor Therapy) were published, focusing on the combination of both morphologic (contemplating a change in the sum of diameters of target lesions according to RECIST 1.1) and metabolic response (i.e., a reduction in the SULpeak >15.5% for the hottest lesion on PET) to assess the clinical benefit of immunotherapy. Clinical benefit includes (a) CR as per RECIST 1.1 (disappearance of all target lesions; reduction in short axis of target lymph nodes to <1 cm; no new lesions); (b) PR as per RECIST 1.1 (decrease in target lesion diameter sum >30%) and (c) SD: Does not meet other criteria plus change in SUL peak of the hottest lesion of ≤15%. No clinical benefit is considered for cases with PMD that include change in SUL peak of the hottest lesion >15% and PD as per RECIST 1.1 (increase in target lesion diameter sum of >20% and at least 5 mm or new lesions) [11].

Last, in 2018, PERCIMT (PET Response Evaluation Criteria for Immunotherapy) was introduced in 2018 for melanoma. The most remarkable change in these criteria is that the appearance of up to four new lesions, depending on their size, can be tolerated to obtain clinical benefit (CB) and support treatment continuation [12]. The categories are (a) CMR: disappearance of all metabolically active lesions; (b) PMR: disappearance of some but not all metabolic lesions and no new lesions; (c) SMD: neither PMD nor PMR/CMR and (d) PMD: 4 or more new lesions (<1 cm in diameter), 3 or more new lesions (>1 cm), 2 or more new lesions (>1.5 cm in diameter).

More recently, other alternative approaches to PERCIST have been used, including iPERCIST [13] and immunotherapy-modified PERCIST5 (imPERCIST) [14]. In the first case, for the iPERCIST criteria, introduction of the immune unconfirmed metabolic progressive disease (iuPMD) acts similarly to iRECIST [15], where subsequent scanning 4–8 weeks is required to confirm or discharge progression. As for imPERCIST criteria, the definition of PMD is reassigned to cases having an increase >30% in the sum of SULpeak of the target lesions (up to 5).

## 4. Distinguishing between “Atypical” Response Patterns

Irruption of immunotherapy in clinical practice has opened very interesting treatment possibilities for oncological patients, but it has, in consequence, meant a new challenge in the field of medical imaging. As mentioned previously, new standardized criteria are needed to evaluate the response to these innovative therapies as their effects on the tumours differ to those conventionally observed with traditional cytotoxic treatments. 

Response criteria for evaluation of solid tumours treated with traditional cytotoxic treatments are focused on reduction or regression of tumour size/burden or decrease in its metabolic volume to categorise a response as favourable. However, immunotherapy causes non-conventional patterns of response. Four new atypical patterns have been described and should be recognized in order to better assess/evaluate a tumour’s response.

### 4.1. Pseudoprogressive Disease (PPD)

Initial enlargement in total tumour volume or onset of new lesions after initiating treatment followed by reduction in tumour burden should be considered pseudoprogression [16]. It is important to highlight that this phenomenon is a reflection of stimulation of the immune response, not linked with real or true progression of a disease. It is caused by infiltration of the tumour environment by the host’s immune activated cells, accompanied by a certain component of oedema, necrosis and haemorrhage [17]. PPD normally takes place within the 4–6 weeks after treatment and can be classified as early or delayed based on time of advent (before or after 12 weeks of therapy). 

PPD was first described in melanoma patients during their treatment with Ipilimumab [18]. In fact, the rate of pseudoprogression is higher in melanoma cases, with up to 10–15% of patients treated with anti-CTLA4 compared to less than 10% of incidence in those treated with anti-PD1 [19]. Its appearance should also be considered in other entities, such as non-small cell lung cancer (NSCLC), renal cancer (RCC), urothelial carcinoma and head and neck squamous cell cancer (HNSCC) among others, although the rate is below 5%. This variance in incidence of pseudoprogression could be related to the idiosyncratic features of the different neoplasms and patients and distinct agents used. There are also publications referring to some particular locations of pseudoprogression [20] and pseudoprogression occurring at different stages of treatment, not just at the beginning.

At this point, it is important to distinguish when we are facing pseudoprogression instead of real progression. The key point should be to check the clinical condition of the patient: clinical improvement should lead to consider pseudoprogression over progression. The checklist proposed by the new guidelines [6] is to be considered a useful help in interpretation and reporting of [^18^F]FDG PET/CT, particularly in case of atypical responses during immunotherapy.

### 4.2. Hyperprogressive Disease (HPD)

**Figure 2:**
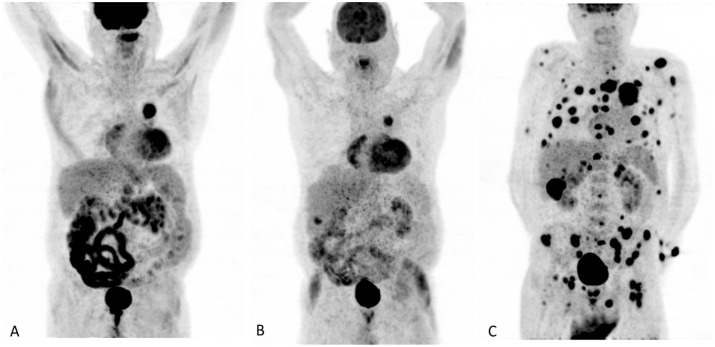
77-year-old man being followed because of an adenocarcinoma in a sigma polyp. He presented an incidental finding of a lung mass located in the superior left lobe, later confirmed as a lung adenocarcinoma with evidence of loco-regional lymphadenopathic infiltration. (**A**) Third [^18^F]FDG PET/CT showing radiological stability after chemotherapy. (**B**) Oligometastatic progression in liver parenchyma with solitary lesion after chemotherapy and radiotherapy. (**C**) After initiating immunotherapy (three cycles of Atezolimumab), [^18^F]FDG PET/CT evidenced progression of the primary tumour and the liver lesion with countless liver, adrenal and bone lytic lesions as well as peritoneal implants, consistent with hyperprogression. The patient died one month later.

Hyperprogression is defined as a considerable and early enlargement of tumour burden following introduction of immunotherapy. An example of hyperprogression is presented in Figure 2. Champiat et al. were the first group to describe this phenomenon in a subset of patients undergoing treatment with anti-PD-1 and anti-PD-L1 [21]. Frequency of hyperprogression varies depending on tumour type and agent used, with rates of incidence within 4–29% in different studies and publications. Nowadays, there is not an established specific criterion to determine recognition of HPD. Consequently, it might be underdiagnosed. It is crucial to diagnose early this abnormal tumour expansion due to its importance in the clinical approach. This scenario leads to readjustment regarding therapy being necessary, including suspension of the active treatment and change to a second line of therapy. In general, it worsens prognoses, with lower global survival rates, and must be considered in patients with high-risk factors, such as elderly, numerous metastatic lesions and history of prior irradiation as well as certain mutations (such as murine double minute 2/4 proto-oncogene (MDM2/4) family amplification or epidermal growth factor receptor (EFGR) aberrations) [22].

### 4.3. Dissociated Response (DR)

**Figure 3:**
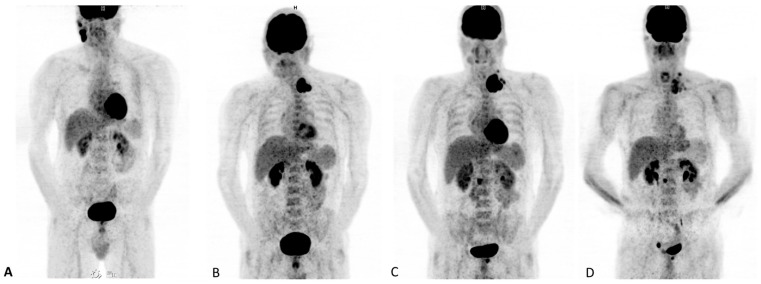
48-year-old man with right temporal melanoma operated in 2016. (**A**) Tumoral progression in laterocervical lymphadenopathies and intraparotid adenopathy in the right side. (**B**) [^18^F]FDG PET/CT 3 months later, under treatment with Dabrafenib and Trimetinib, shows a complete response in the right side with lymphadenopathic progression on the left side, where a new conglomerate is identified. (**C**) Control after one month shows lymphadenopathic progression and two new lesions in lumbar spine and pubis. (**D**) After 4 cycles of Nivolumab and Ipilimumab, there is improvement in cervical lymph node involvement, while bone progression is observed in pelvis. This evolution suggests dissociated response.

Growth of certain lesions accompanied by the paradoxical shrinkage in baseline lesions should be classified as a dissociated response. An example of dissociated response is presented in Figure 3. It could also be reported as mixed response or disproportional response [23]. This is not really a novel pattern, having already been identified with traditional treatments (as platinum-based chemotherapy). DR has been described in different studies, with a rate not overcoming 10% overall [23,24,25]. Interestingly, onset of DR has showed a potential association with favourable prognosis in comparison with patients developing a homogeneous progression. This subset of patients might obtain a benefit better by not discontinuing initial immunotherapy treatment. In addition, it is relevant to identify oligometastatic patients who may benefit from local therapies [26].

### 4.4. Sustained Response (SR)

Immunotherapeutic agents are superior to conventional drugs due to their ability to induce enduring responses despite having completed the treatment. This manifestation has been observed in 10–25% of metastatic patients. According to classical criteria (RECIST or WHO criteria), the lack of either partial or complete radiological response was to be classified as treatment failure, with subsequent categorization of those patients as non-responders [27]. Pons-Tostivint et al. demonstrated superiority of immune checkpoint inhibitors over other systemic treatments in terms of durable responses and overall survival. They also identified a major proportion of sustained response in the group of patients treated with anti-PD-1/PD-L1 agents [28].

## 5. Application of PET Biomarkers as Predictive and Response Evaluation Parameters

Application of PET biomarkers as predictive and response evaluation parameters in evaluation of melanoma patients treated with immunotherapy has been studied in recent publications. [^18^F]FDG PET/CT imaging biomarkers can be classified based on three aspects: tumour burden, tumour glucose uptake and nontumoral hematopoietic tissue metabolism. The first group (tumour burden) includes three measures: metabolic tumour volume (MTV), total MTV (TMTV) and total lesion glycolysis (TLG). The second group (tumour glucose uptake) includes three measures: maximum standard uptake value (SUVmax), standardized uptake value corrected for lean body mass (SUL) and heterogeneity index of SUV (HISUV). The third group (nontumoral hematopoietic tissue metabolism) includes parameters focusing on medullary and extra-medullary haematopoiesis, such as spleen-to-liver maximum standard uptake value ratio (SLR) and bone marrow-to-liver maximum standard uptake value ratio (BLR) [29]. 

A recent meta-analysis by Ayati et al. [4] analysed patients with metastatic melanoma treated with immunotherapy, investigating the role of [^18^F]FDG PET/CT for predicting and monitoring immunotherapy response regardless of the kind of immunotherapy employed. This meta-analysis included 24 articles published between October 2014 and June 2020. They concluded that three of the parameters most used in PET, being MTV, TLG and SUL/SUV peak, could be a convenient tool to predict response in patients with metastatic melanoma. Regarding their analysis of [^18^F]FDG PET biomarkers, Ayati et al. divided the selected articles into two groups: one centred on the baseline [^18^F]FDG PET/CT parameters and the other focused on the metabolic changes between baseline and follow-up [^18^F]FDG PET/CT. In the first group that analysed baseline [^18^F]FDG PET/CT parameters for prediction of outcomes, the studies included found that MTV and TLG were associated with overall survival (OS) and progression-free survival (PFS) rates in most studies. The second group analysed the value of interval changes in baseline and late [^18^F]FDG PET/CT parameters as predictor of outcomes, taking into account clinically oriented indexes. The studies included reported that changes in SUVmax were not associated with differences in the outcomes. However, one study [12] found that the absolute number of new focal hypermetabolic lesions was a better marker than changes in SUVmax.

Regarding the individual studies published in this field, we have summarised the main findings of 13 original articles published since 2017 in Table 1 [11,12,29,30,31,32,33,34,35,36,37,38,39], presenting their main characteristics, endpoint and results. Most articles focus on the baseline [^18^F]FDG PET/CT and just a few articles focus on the interval changes between the baseline and follow-up [^18^F]FDG PET/CT.

### 5.1. Analysis Focused on the Baseline [^18^F]FDG PET/CT

This first group, which analysed baseline [^18^F]-FDG PET/CT parameters for prediction of outcomes, included thirteen studies [11,12,29,30,31,32,33,34,35,36,37,38,39]. The [^18^F]FDG PET/CT parameters analysed were MTV, BLR, SLR, SUVmax, SUVpeak, TLG and tumour heterogeneity index.

Up to now, MTV is the main parameter showing prognostic value. Ito et al. [30] found a significant correlation of MTV and TLG with OS as well as concluding that TMTV may be a strong independent prognostic factor. These same parameters were analysed in the study by Nakamoto et al. [33] published in 2020, which reported a significant correlation with OS. In the study by Seban et al. published in 2019 [29], MTV also correlated with OS, concluding that a low tumour burden correlated with survival and objective response. They also evaluated nontumoral hematopoietic tissue metabolism, finding that not only TMTV but also BLR had significant and independent prognostic value, correlating inversely with OS and PFS. A possible explanation for the fact that increased metabolism in bone marrow can correlate with worse outcomes is that the bone marrow has cells relevant to some tumour formation mechanisms, such as neovascularization and priming of metastases [40,41].

The study published in 2021 by Nakamoto et al. [37] reported that BLR was an independent prognostic biomarker for OS and PFS. A stratified analysis, combining BLR with independent clinical factors with three categories, found a worse OS in the group with higher BLR and unfavourable clinical risk factors. Regarding BLR and MTV, there was a weak (0, 34) positive correlation between both. As in previous studies [29,30], MTV was associated with OS.

Flavus et al. [36], who reported a correlation between MTV with OS, additionally analysed textural PET parameters using the radiomics with the same purpose of predicting outcomes. Forty-one image biomarker standardization initiative (IBSI)-compliant parameters were studied and only long zone emphasis (LZE) correlated with shorter OS, the same as MTV. Both parameters, MTV and LZE, were used to perform a prognostic score in which patients were categorized into three groups.

On the contrary, Sanli et al. [31] did not find correlation between MTV and OS. However, SUVmax, SUVpeak and TLG were associated with OS. Another parameter analysed, intratumoral metabolic heterogeneity, measured using the tumour heterogeneity (TH) index, also showed significant association with OS.

Furthermore, SLR, a nontumoral hematopoietic tissue metabolism parameter, was analysed by Wong et al. [35] and Seban et al. [29,34]. Wong et al. reported that an SLR greater than 1.1 is associated with a poor outcome (OS and PFS) after ipilimumab but not after PDL-1. On the contrary, Seban et al. [29,34], did not find a significant association between SLR and OS. Regarding other parameters, Wong et al. [35] reported that SUVmax was not associated with OS or PFS, whereas MTV was only significantly associated with OS when it was analysed as a continuous variable.

Another study analysing SLR, by Sachpekidis et al. [38], differed from the previous ones described in that it evaluated early disease progression and immune activation related to confirmed progressive metabolic disease versus pseudoprogression instead of survival rates. Patients categorised as confirmed progressive metabolic disease showed higher SLRmean after the first two cycles of immunotherapy than those catalogued as pseudoprogression. In the analysis of SLRmean and SLRmax in baseline PET, there were no significant differences between patients classified as confirmed progressive metabolic disease and those classified as pseudoprogression. These results suggest that a higher SLR may be associated with a poor clinical outcome, as Wong et al. [35] had also highlighted.

Nobashi et al. [32] analysed patients not only with melanoma but also with malignant lymphoma and renal cell carcinoma, finding no statistical differences between patients with and without clinical benefit and baseline SUVmax, MTV and TLG. On the other hand, in patients with clinical benefit, a significant decrease in PET parameters (SUVmax, MTV and TLG) of the first restaging PET/CT was observed, but not in patients with no clinical benefit.

Finally, Schweighofer-Zwink et al. [39] reported that metabolic parameters and tumour-to-background ratios (TBRs) were correlated with OS, not only in the baseline [^18^F]FDG PET/CT but also in the two follow-up [^18^F]FDG PET/CT scans, performed 3 and 6 months after immunotherapy. In the baseline [^18^F]FDG PET/CT, SULmax and SULpeak as well as most of TBRs were predictive for 3-year and 5-year OS rates. In the follow-up studies, MTV, TLG and most of the TBRs were predictive. On the other hand, changes in values of MTV, TLG and most of the TBRs from the baseline PET to the follow-up studies were prognostic.

### 5.2. Analysis Focused on the Interval Changes between Baseline and Follow-Up [^18^F]FDG PET/CT

The second group, which analysed the value of interval changes in baseline and late [^18^F]FDG PET/CT parameters as predictor of outcomes, took into account clinically oriented indexes. Two articles evaluated changes between baseline [^18^F]FDG PET/CT and after starting immunotherapy using patients’ clinical response as reference. These studies, by Cho et al. [11] and Anwar et al. [12], were used as the basis to propose the PECRIT and PERCIMT, respectively. Regarding changes in SUVmax, no correlation with clinical response was observed, as described by Anwar et al. [12], who concluded that number of new lesions in PET may be a good response marker. 

When considering follow-up in patients undergoing treatment with checkpoint inhibitors for one year [42], use of metabolic information enabled better prediction of long-term benefit regardless of partial responses on morphological imaging. Moreover, five years after the 1-year PET in melanoma treated with anti-PD-1 therapy [43], [^18^F]FDG PET/CT could still predict progression better than CT, especially in those patients with residual disease on CT.

In summary, [^18^F]FDG PET/CT biomarkers could be a promising parameter to predict outcome and assess response in these patients. Earlier prediction would provide the information necessary to adapt the treatment, while later assessment before ceasing treatment [6] can help discontinue more safely immunotherapy. Therefore, in the near future, further evidence may support personalising patient management based on these biomarkers.

## 6. Diagnosis and Management of Immunorelated Adverse Effects

Checkpoint inhibitors used in immunotherapy can cause inflammation of any tissue or organ, being responsible for immune-related adverse events (irAEs). With the increasing use of immunotherapy in clinical practice, irAEs are increasing as well. Immune-related toxicities vary in terms of their time of onset, severity, underlying biology and the way of use, either in monotherapy or combined therapy, which are often used in advanced or higher risk diseases [4].

Severity of irAEs is characterized by grades [44], ranging from grade 1 to 5: (a) Grade 1–2: Include situations with very manageable symptoms, which can be treated just with corticosteroids. (b) Grade 3: Cases with moderate/severe symptoms, which will need to stop immunotherapy treatment and undergo hospitalization to be controlled and treated. (c) Grade 4: Life-threatening situations that, although rare, are more common with anti-PD-1/PDL-1 treatments and in combination therapy than in monotherapy. It is important for clinicians to be aware of these life-threatening adverse events in order to start early treatment. (d) Grade 5: Fatal irAEs include neurotoxicity, cardiotoxicity and pulmonary toxicity. A summary of the categorization of immune-related adverse events (irAEs) based on severity is presented in Table 2.

The main characteristics of irAEs are (a) typical onset is within 2–16 weeks but can occur at any time after receiving immunotherapy treatment (from days to even after a year); (b) each irAE can become serious if not diagnosed early and appropriately treated; (c) most symptomatic irAEs, except those involving the endocrine system, are managed by withdrawal of the treatment, if needed, plus several weeks of glucocorticoid treatment. Most irAEs resolve with no further actions, but some require chronic therapy (hormonal supplementation, immunosuppression treatment, etc.). Those that affect the endocrine system should be studied with serum markers. Patients may be asymptomatic but may still require modifications of their treatment or steroid therapy [45].

What is the role of [^18^F]FDG PET/CT in diagnosis and management of immunorelated adverse effects? [^18^F]FDG PET/CT is a great tool in clinical practice to detect early signs of irAEs, such as tissue inflammation, which will enable a clinician to intervene even before the symptoms appear. Nuclear medicine physicians must be aware of these potential artefacts and the spectrum of potential non-malignant inflammatory changes in patients with immunotherapy treatment to avoid diagnostic mistakes [46]. Below are described the most common irAEs known in melanoma treatment with immunotherapy.

Another potential role of [^18^F]FDG PET/CT in this setting its predictive value when iRAEs are detected. However, up to now, the available evidence is non-conclusive [4]. For example, in one study, they found significant correlation of irAEs with therapy response for some irAEs, such as hypophysitis, hepatitis, skin rash, pruritus, fever and ocular muscle inflammation (*p* < 0.05), but, on the contrary, they did not find any significant correlation between PET-related colitis or diarrhoea and response to therapy (*p* > 0.05) [47]. It has been observed that presence of irAEs, particularly severe irAEs, correlates with response to immunotherapy, disease control and good long-term survival [47,48]. However, patients without any irAEs or only mild irAEs also reached similar outcomes, Because of this, presence of irAEs should not be considered an essential condition for achieving clinical benefit [33,47]. In this regard, occurrence or severity of irAEs should not be the basis of decisions to continue or cease immunotherapy [48]. Other aspects to be considered regarding irAEs are that there are sex-specific differences (i.e., endocrinological IRAEs more often in women) and potential differences in survival between males and females [4]. Finally, management of IRAEs during treatment might also be challenging, specifically regarding the decision of discontinuation of treatment with immunotherapy [4].

### 6.1. Nodal Activation and Sarcoid-like Reaction

The differential diagnosis of lymphadenopathies is very long. In the context of melanoma’s immunotherapy, lymphadenopathies located in the draining basins from the site of the tumour can be a challenging issue as it is difficult to differentiate between reactive nodes from metastatic disease. Some of the signs that allow clinician to differentiate between both diagnoses are (a) the size and shape of the nodes; those that are round with a short to long axes ratio (S/L ratio) greater than 0.5) are suggestive of malignant disease, while reactive or benign lymph nodes are elliptical in shape (S/L ratio <0.5); (b) preservation of the nodal fatty hilar structure preserved, low–mild [^18^F]FDG metabolism, symmetrical [^18^F]FDG uptake are highly suggestive of benign lesions [46,49].

**Figure 4:**
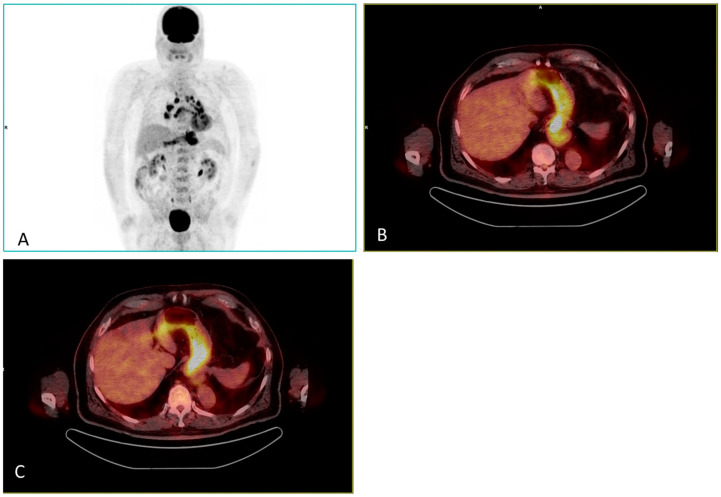
73-year-old man with dorsal melanoma under treatment with Nivolumab and granulomatous reaction sarcoid-like as well as acute gastritis. [^18^F]FDG PET/CT MIP (**A**) and axial fusion slices (**B**,**C**) are presented. Treatment was discontinued.

On the other hand, sarcoid-like reactions have been related with PD-1 anti-neoplastic immunotherapy against melanoma. This irAE mimics this multisystem granulomatous disease, appearing as a systemic granulomatous reaction that is indistinguishable from sarcoidosis. Some signs that enable a clinician to identify it as a sarcoid-like reaction is that it typically appears after initiation of treatment and it improves or resolves after withdrawal of treatment. [^18^F]-FDG PET/CT shows symmetrical multiple foci uptake observed in the mediastinal and bilateral hilar nodes, but it can also be seen in retrocrural and abdominal para-aortic nodes. However, to distinguish between sarcoidosis occurring in an oncologic patient and sarcoid reactions is difficult unless a pathology study is performed (granulomas in sarcoid reactions are B-cell positive, whereas those in sarcoidosis are B-cell negative) [46,49]. An example is presented in Figure 4.

### 6.2. Reactive Bone Marrow

This may happen when the oxygen content in the body tissues is low, if there is loss of blood or anaemia or if the number of red blood cells decreases, but it also may happen as a reaction to immunotherapy agents that work as activators of the immune system. It may involve any bone, but the predominant sites include the vertebral column, ribs, skull, pelvis, etc. Diffuse homogeneous [^18^F]-FDG uptake is observed on [^18^F]FDG PET/CT, which reflects hyperplastic bone marrow and an activated immune system [46].

### 6.3. Splenic Activity

It indicates activation of reticuloendothelial system promoted by immunotherapy agents. High diffuse [^18^F]FDG uptake is observed in splenic tissue, which is higher than the liver uptake (as inversion of the usual liver-to-spleen uptake ratio) regardless of whether or not there is splenomegaly [46]. 

### 6.4. Thyroiditis

The clinical manifestations can range from hypothyroidism to hyperthyroidism. To achieve proper diagnosis, correlation with clinical and hormonal analysis will be needed. 

These irAEs are much more frequent when related to PD-1 immunotherapy against melanoma. Diffuse uptake of [^18^F]FDG in the thyroid gland is observed on [^18^F]FDG PET/CT, commonly related to benign processes [46].

### 6.5. Pneumonitis

Pneumonitis is a rare but severe immune-related adverse event. It is considered a stage 4-degree severity, so clinicians that evaluate these patients must be aware and initiate an evaluation for pneumonitis when the first signs or symptoms appear, such as cough, fever, dyspnoea or chest pain, and, once the diagnosis is confirmed, pulmonary function should be monitored serially to evaluate for progression or resolution of pneumonitis. It presents four distinct patterns: organizing pneumonia (OP); nonspecific interstitial pneumonia (NSIP); hypersensitivity pneumonitis (HP); diffuse alveolar damage (DAD) [50].

### 6.6. Colitis

It is described as diarrhoea that requires steroid/infliximab therapy for resolution and/or with endoscopic/histological confirmation (colonoscopy and biopsy are the gold standard for diagnosis in this situation). There is a significantly higher risk of developing colitis with combined immunotherapy treatment. Disease severity can range from grade 1 to 4 depending on its symptoms, which are summarised in Table 3. 

Parched uptake with moderate to high [^18^F]FDG uptake is shown in the colon on [^18^F]FDG PET. It should be noted that use of metformin can result in important increased uptake of [^18^F]FDG and should be ceased at least 48 h prior to [^18^F]FDG PET/CT [51,52].

### 6.7. Hepatitis

It is a potentially serious complication of checkpoint blockade. Hepatitis is most commonly a low-grade toxicity, but grade 3 and 4 hepatotoxicity does occur; its incidence is especially high for combined immunotherapy. An increase in liver markers is a sign for imminent severe disease (although level of transaminases does not always correlate with histologic extent of injury). Signs of severe liver injury should be evaluated (asterixis, ascites, caput medusa, hepatomegaly, jaundice, scleral icterus) although hepatitis from immunotherapy agents is not usually detectable in physical examination. Generally, [^18^F]FDG PET/CT does not show significant metabolic alterations in the liver [46].

### 6.8. Pancreatitis

The incidence of pancreatitis with either of the inhibitors is low. It is generally associated with a rise in serum amylase but may be clinically asymptomatic, showing a decrease in endocrine and exocrine pancreatic function, which result in metabolic and nutritional disorders. Signs that must alarm the clinician towards pancreas injury are hyperglycaemia, abdominal pain and steatorrhea. On [^18^F]FDG PET/CT, the pancreas may present diffuse [^18^F]FDG uptake of moderate to high intensity in addition to focal or diffuse pancreatic enlargement without a focal lesion suspicious for metastasis [46].

### 6.9. Hypophysitis

It is predominantly a complication of CTLA-4 inhibitors, although the mechanism by which hypophysitis occurs after CTLA-4 inhibitors exposure is not clear. Most patients remain on glucocorticoid replacement despite efforts to withdraw therapy and few patients fully recover pituitary–adrenal axis function. Men are more prone to developing immunorelated hypophysitis than women. Clinicians should expect notable increases in incidence and prevalence of hypopituitarism secondary to hypophysitis after immune checkpoint inhibitor. On [^18^F]FDG PET/CT, hypophysitis is shown as a discernible focal new [^18^F]FDG uptake in the pituitary fossa [46].

### 6.10. Skin and Soft Tissue

This is the most common of all irAEs, especially with combined therapy. Clinical manifestations range from pruritus and mild dermatoses to severe reactions, including Stevens–Johnson syndrome and toxic epidermal necrolysis. On [^18^F]FDG PET/CT, skin manifestations are generally not visualized, but subcutaneous tissue or panniculitis can be visualized as nodules with moderate [^18^F]FDG avidity within areas of subcutaneous fat [46].

## 7. Discussion

This review is focused on melanoma patients, analysing (a) the role of [^18^F]FDG PET/CT in the above mentioned challenges; (b) the available evidence on its efficacy. For this purpose, we performed a review of the literature, focusing on original and review articles. Immunotherapy is based on manipulation of the immune system in order to act against tumour cells, with growing evidence especially in melanoma patients. The available evidence suggests that [^18^F]FDG PET/CT has prognostic value in melanoma patients treated with immunotherapy.

The first challenge faced by this new therapeutic tool is finding valid evaluation criteria for response assessment. To standardize the evaluation criteria in order to be able to apply them in clinical practice for evaluation of response to immunotherapy, new evaluation criteria have been published. In summary, although there are no clearly established or globally accepted criteria, modified response criteria are potentially an appropriate method for evaluation of immunotherapy benefit. These modified response criteria, focusing on the metabolic response, are potentially appropriate for evaluation of response to immunotherapy. These criteria consider the fact that immunotherapy can cause non-conventional patterns of response and must be considered for precise evaluation.

The second challenge is distinguishing between “atypical” response patterns. In this regard, immunotherapy causes non-conventional patterns of response. Four new atypical patterns have been described and should be recognized in order to better assess/evaluate a tumour’s response.

The third challenge is using PET biomarkers as predictive and response evaluation parameters, divided into two categories: those obtained from the baseline study and those that result from interval changes. In this context, [^18^F]FDG PET/CT biomarkers appear to be promising parameters in prediction and assessment of response to immunotherapy.

The fourth and last challenge is diagnosis and management of immunorelated adverse effects. Immunorelated adverse effects are recognized as predictors of early response to immunotherapy and may be associated with better prognosis and clinical benefit.

The main limitation of this study is it is not a systematic review. However, a recent meta-analysis was updated with studies published since the literature search was completed. The main findings of our review are in line with those of the systematic review. 

The available evidence suggests that [^18^F]FDG PET/CT has a prognostic tool in melanoma patients treated with immunotherapy. However, there are no globally accepted response criteria yet and the evidence is scarce. Therefore, new studies are warranted in order to obtain high-quality evidence.

## Figures and Tables

**Table 1:** Characteristics of the articles included and main findings.

Author	Year	Design	Sample Size	Type of Immunotherapy	PET Parameters	Summary Main Findings
Cho et al. [11]	2017	Pros. *	20	Ipilimumab nivolumab	SUV	No statistically significant differences between SUVmax in basal and late PET
Anwar et al. [12]	2018	Pros. *	41	Ipilimumab	SUV	No statistically significant differences between SUVmax in basal and late PET
Ito et al. [30]	2019	Retr. #	142	Ipilimumab	MTV, TLG	TMTV was a strong independent prognostic factor
Sanli et al. [31]	2019	Retr. #	34	Anti-PD1	SUV, MTV, TLG, TH index	Analysis showed that SUVmax, SUVpeak, gradient-based TLG and gradient-based TH index had a significant association with OS. There was no correlation between MTV and OS
Seban et al. [29]	2019	Retr. #	55	Anti-PD1	SUV, MTV, TLG, HISUV, BLR, SLR	Low tumour burden (MTV) correlates with survival and objective response. Hematopoietic tissue metabolism (BLR) correlates inversely with survival
Nobashi et al. [32]	2019	Retr. #	40	Ipilimumab, pembrolizumab, nivolumab	SUV, MTV, TLG	There was no statistical difference for baseline SUVmax, MTV nor TLG between patients with and without clinical benefit
Nakamoto et al. [33]	2020	Retr. #	85	Ipilimumab, pembrolizumab, nivolumab	MTV, TLG, SUV	TMTV was a strong prognostic indicator of OS in melanoma patients
Seban et al. [34]	2020	Retr. #	56	PD-1, CTLA-4	SUV, MTV, TLG, HISUV, BLR, SLR	For mucosal melanoma patients, the only prognostic imaging biomarker was SUVmax, whereas, for cutaneous melanoma patients, MTV, TLG and BLR were negatively correlated to ICI response duration
Wong et al. [35]	2020	Retr. #	90	Ipilimumab or anti-PD1	SUV, MTV, SLR	Pre-treatment SLR > 1, 1 was associated with poor outcome after ipilimumab
Flavus et al. [36]	2021	Retr. #	56	Ipilimumab, pembrolizumab	SUV, MTV, LZE	Total MTV and LZE correlated with shorter OS
Nakamoto et al. [37]	2021	Retr. #	92	Ipilimumab, pembrolizumab	SUV, MTV, TLG, BLR	BLR was an independent prognostic biomarker for OS and PFS; high BLR was associated with poor progression-free and overall survival
Sachpekidis et al. [38]	2021	Retr. #	31	Ipilimumab, nivolumab	SLR	Patients catalogued as confirmed progressive metabolic disease had higher SLRmean after 2 cycles of treatment than those catalogued as pseudoprogression
Schweighofer-Zwink et al. [39]	2021	Retr. #	51	Ipilimumab, pembrolizumab, nivolumab	SUL, MTV, TLG, TBR of SUL	On baseline, PET, SULmax and SULpeak as well as most TBRs were predictive for 3- and 5-year OS rates. MTV, TLG and most of the TBRs were predictive on both follow-up studies (3 and 6 months after therapy). Changes in values of MTV, TLG and most of the TBRs from the baseline to the follow-up studies were prognostic

* Prospective design; # Retrospective design. BLR: Bone marrow-to-liver maximum standard uptake value ratio; HISUV: Heterogeneity index of SUV; ICI: Immune checkpoint inhibitor; LZE: Long zone emphasis; MTV: Metabolic tumour volume; SLR: Spleen-to-liver maximum standard uptake value ratio; SLRmean: Mean spleen-to-liver maximum standard uptake value ratio; SUL: Standardized uptake value corrected for lean body mass; SULmax: Maximum standardized uptake value corrected for lean body mass; SULpeak: Peak standardized uptake value corrected for lean body mass; SUV: Standard uptake value; SUVmax: Maximum standard uptake value; SUVpeak: Peak standard uptake value; TBR: Tumour-to-background ratio; TH index: Tumour heterogeneity index; TLG: Total lesion glycolysis; TMTV: Total MTV.

**Table 2:** Categorization of immune-related adverse events (irAEs) based on severity.

Grade	Definition
Grade 1	Mild
Grade 2	Moderate
Grade 3	Severe or requiring hospitalization but not life-threatening
Grade 4	Life-threatening
Grade 5	Death

**Table 3:** Symptoms and management of colitis according to grade. Table adapted from Som A. et al. [51,52].

Severity	Symptoms	Management
Grade 1	Asymptomatic	Close monitoring immunotherapy. Loperamida/Difenoxilato/atropine
Grade 2	Abdominal pain, mucus, blood in stool	Systemic steroids (if no response in 2–3 days, consider adding infliximab within 2 weeks)
Grade 3	Severe abdominal pain, peritoneal signs	Require hospitalization for supportive care: - IV corticosteroids. - If no response in 2 days, strongly consider adding infliximab or vedolizumab within 2 weeks.
Grade 4	Severe and persistent abdominal pain, fever, ileus, life-threatening complications, such as perforation and peritonitis.	

## Data Availability

Not applicable.
